# Unraveling Acute Kidney Injury: An Intricate Case of Sepsis and Immune-Mediated Renal Damage

**DOI:** 10.7759/cureus.87373

**Published:** 2025-07-06

**Authors:** Maha Hassan, Melissa Perez, Michael Girdler, Amarbir Dhillon, Prashant Karkal

**Affiliations:** 1 Family Medicine, Larkin Community Hospital Palm Springs Campus, Hialeah, USA; 2 Medicine, Nova Southeastern University Dr. Kiran C. Patel College of Osteopathic Medicine, Fort Lauderdale, USA

**Keywords:** acute kidney injury care, anca negative, elevated creatinine, immune-mediated aki, pauci-immune-mediated aki

## Abstract

Acute kidney injury (AKI) is a critical condition characterized by a sudden decline in kidney function, often posing diagnostic and therapeutic challenges due to its multifactorial nature. This case is significant, as it exemplifies the complexity of AKI in the context of coexisting sepsis, potential antibiotic nephrotoxicity, and immune-mediated processes. While AKI related to sepsis and renal toxic antibiotics is well-documented, the rapid onset and the involvement of antineutrophil cytoplasmic antibody (ANCA)-negative pauci-immune glomerulonephritis add a unique dimension to this case, contributing novel insights to the medical literature. ANCA-negative pauci-immune glomerulonephritis is characterized by a rapidly progressive glomerulonephritis that lacks the detection of antineutrophil cytoplasmic antibodies.

We report the case of a 68-year-old Hispanic male with a history of asthma and pulmonary emphysema, presenting with acute respiratory symptoms, including shortness of breath, chills, and a productive cough, accompanied by gastrointestinal symptoms. The patient was admitted to the hospital with pneumonia complicated by sepsis. Initial treatment included antibiotics known for potential renal toxicity. Within 48 hours, the patient experienced a rapid deterioration in renal function, marked by a significant increase in serum creatinine levels. A renal biopsy revealed acute tubular injury with no immune complex deposition and an elevated myeloperoxidase level, suggestive of an immune-mediated process. The patient responded positively to immunosuppressive therapy, consisting of rituximab and corticosteroids, resulting in improved renal function.

This case underscores the importance of considering a broad differential diagnosis in AKI, particularly in patients with complex clinical presentations. The involvement of ANCA-negative pauci-immune glomerulonephritis highlights the need for awareness of immune-mediated renal injuries in similar clinical scenarios. This case has implications for both nephrology and broader medical practice, as it emphasizes the necessity of integrating clinical, laboratory, and histopathological data to guide effective treatment strategies. The findings advance our understanding of the potential interplay between sepsis, drug-induced nephrotoxicity, and immune-mediated processes in AKI, offering valuable insights for future diagnostic and therapeutic approaches.

## Introduction

Acute kidney injury (AKI) is characterized by a rapid decline in renal function, clinically demonstrated by a rapid increase in serum creatinine levels and/or sharp reduction in urine output. In non-hospitalized patients, the most prevalent cause of AKI is decreased renal perfusion, whereas in hospitalized patients, the leading cause of AKI is acute tubular necrosis [1]. On the other hand, AKI may result from sepsis and systemic inflammatory conditions, driven by various factors, including the kidneys’ critical role in clearing and filtering circulating cytokines and bacterial toxins [2]. 

In this case report, we present a 68-year-old male with a complex medical history, including asthma, pulmonary emphysema, a three-month history of non-productive cough, and a 50-year pack smoking history who presented to the ED with new onset shortness of breath (SOB), chills, nausea, vomiting, a single episode of diarrhea, and a five-day history of productive cough. During the ED evaluation, the patient experienced an episode of gross hematuria with rapid clot formation, which precluded urine analysis evaluation. Subsequently, beginning on the second day of hospitalization, the patient’s serum creatinine level began to rise from a baseline of 1.38 mg/dL to 5.02 mg/dL on the third day of admission, eventually peaking at 12.08 mg/dL on the fourteenth day after admission. This case underscores the diagnostic complexity of AKI in the context of multifactorial etiologies and highlights the importance of a comprehensive and systematic approach to its evaluation and management.

## Case presentation

A 68-year-old Hispanic male with a past medical history of asthma, pulmonary emphysema, a three-month history of non-productive cough, and a 50-year pack smoking history presented to the ED due to new onset SOB, chills, nausea, vomiting, diarrhea, and a five-day history of productive cough. Two days before presenting to the ED, he was evaluated by his primary care provider, who prescribed him amoxicillin 500 mg every 8 hours, benzonatate 100 mg every 8 hours, and albuterol sulfate 90 mcg inhaler, two puffs every 6 hours. He had taken three doses of amoxicillin without improvement of the symptoms at the time of evaluation in the ER. 

Upon arrival, the patient presented with a blood pressure of 169/86 mmHg, a temperature of 99.2°F, a respiratory rate of 24 bpm, a heart rate of 117 bpm, and an oxygen saturation of 92%. On physical examination, he exhibited mild crackles in the right lower lobe on respiratory examination, and there were no signs of periorbital edema or pitting edema. He appeared well-developed and well-nourished and was alert and oriented to person, place, and time. Laboratory findings revealed leukocytosis with left shift (WBC: 31.8×10⁹/L and neutrophils: 88%), lactic acid: 3 mmol/L, hemoglobin: 10.7 g/dL, creatinine: 1.38 mg/dL, and potassium: 4.5 mmol/L. Additionally, infectious workup for *Mycoplasma*, *Legionella*, influenza A and B, and COVID-19 were all negative (Table 1).

**Table 1:** ER laboratories

ED laboratories		
Comprehensive metabolic panel	Reference	Patient’s results
Sodium	137-145 mmol/L	134
Potassium	3.5-5.1 mmol/L	4.5
Chloride	98-107 mmol/L	109
Glucose	74-106 mg/dL	128
Blood urea nitrogen	9-20 mg/dL	23
Creatine	0.66-1.25 mg/dL	1.38
Estimated glomerular filtration rate	>60 mL/min/1.73 m^2^	51
Alanine aminotransferase	0-35 U/L	29
Aspartate aminotransferase	15-46 U/L	84
Total bilirubin	0.2-1.3 mg/dL	6.8
Direct bilirubin	0.0-0.3 mg/dL	3.1
Lactic acid	0.7-2.0 mmol/L	3.0
Complete blood count		
White blood count	3.4-11 10*3/uL	31.8
Hemoglobin	13-17.2 g/dL	10.7
Platelets	130-360 10*3/uL	426
Neutrophil	40%-70%	88%
COVID-19	Non-reactive	Non-reactive
Mycoplasma	Non-reactive	Non-reactive
Influenza A/B	Non-reactive	Non-reactive
Legionella	Non-reactive	Non-reactive

**Figure 1:**
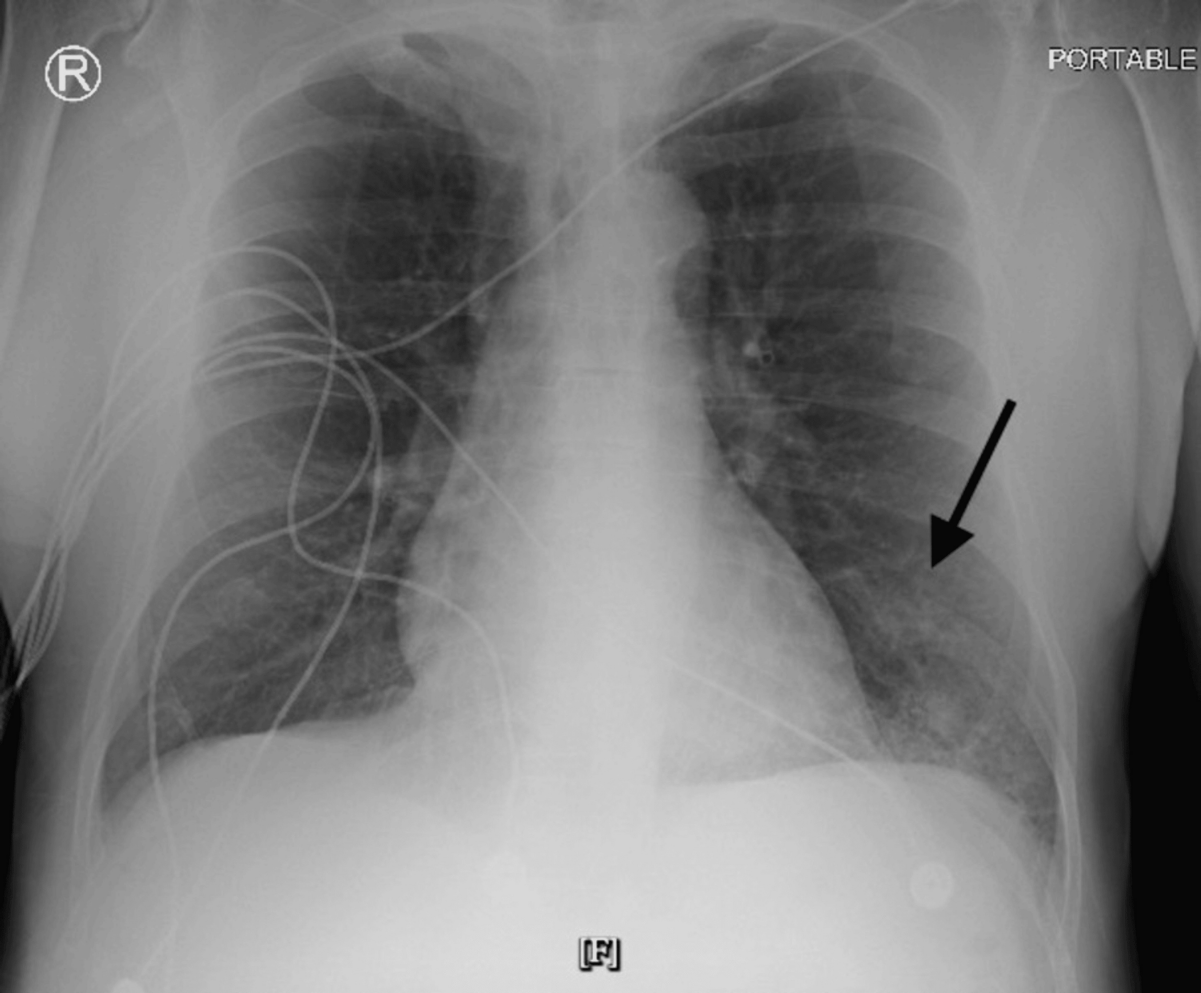
ED chest X-ray

Radiologic imaging included a chest X-ray, which revealed patchy opacities in the left lung base, consistent with pneumonia (Figure 1). Based on the clinical, laboratory, and radiologic findings, the patient was diagnosed with pneumonia complicated by sepsis without septic shock; after blood and sputum cultures were obtained, antibiotic therapy was initiated with vancomycin 1 gm and piperacillin-tazobactam 4.5 gm, followed by the addition of azithromycin 500 mg. Approximately one hour after the initiation of the antibiotics, he reported an episode of gross hematuria with rapid coagulation, which precluded the performance of a urine analysis. The patient was admitted to the hospital for inpatient management and evaluation. The patient's CURB-65 score was 2, corresponding to moderate risk.

Forty-eight hours after admission, the patient’s creatinine, blood urea nitrogen (BUN), and glomerular filtration rate (GFR) levels sharply differed from a baseline of 1.38 mg/dL, 23 mg/dL, 51 mL/min/1.73 m^2^ to 5.02 mg/dL, 48 mg/dL, and 12 mL/min/1.73 m^2^, respectively (Table 2).

**Table 2:** Creatinine, GFR, and BUN levels during hospital admissions *Day 14: Hemodialysis started BUN, blood urea nitrogen; GFR, glomerular filtration rate

	Reference	Day 2	Day 7	Day 14*	Day 26
Creatinine	0.66-1.25 mg/dL	5.02	10.96	12.08	3.11
GFR	>60 mL/min/1.73 m^2^	12	5	6	20
BUN	9-20 mg/dL	48	77	87	58

Due to the patient’s deterioration of renal function, nephrology was consulted who initiated a work-up that included levels of complement component three, complement component four, perinuclear ANCA, ANCA, cytoplasmic ANCA, myeloperoxidase (MPO), the workup was remarkable for MPO at 673 pmol/L as seen in Table 3. 

**Table 3:** Autoimmune panel work-up C3, complement component 3; C4, complement component 4; c-ANCA, cytoplasmic antineutrophil cytoplasmic autoantibody; MPO, myeloperoxidase; p-ANCA, perinuclear anti-neutrophil cytoplasmic antibody

Autoimmune panel	Reference	Patient's results
C3	82-167 mg/dL	120
C4	12-38 mg/dL	31
p-ANCA	Neg: 1:20	<1:20
c-ANCA	Neg: 1:20	<1:20
MPO	0-469 pmol/L	673

Additionally, a renal ultrasound demonstrated an unremarkable gross appearance of the kidneys.

Despite supportive measures such as discontinuation of renal toxic antibiotics and intravenous fluid administration, his renal function continued to deteriorate with creatinine, BUN, and estimated GFR levels reaching a peak of 12.08 mg/dL, 87 mg/dL, and 6 mL/min/1.73 m^2^ on the fourteenth day of hospitalization (Table 2). On the fourth day after admission, a tunneled dialysis catheter was placed in the right internal jugular vein, and hemodialysis was started.

A renal biopsy was performed on the sixth day after admission. The pathology results were consistent with the following: “acute tubular injury and numerous red blood cell casts, in the absence of immune complex deposition and crescentic glomerulonephritis. The differential diagnosis included unsampled glomerulonephritis pauci-immune type”. The possibility of an atypical ANCA may be considered. Additionally, anticoagulation-related nephropathy can yield similar histopathological findings.

Therefore, given that an underlying immune-mediated renal injury was considered as a possible culprit of AKI, high-dose solumedrol 500 mg IV daily for three days, followed by prednisone 60 mg daily for another three days, was initiated on the tenth day of admission. Subsequently, rituximab was added to the patient's treatment plan. 

**Figure 2:**
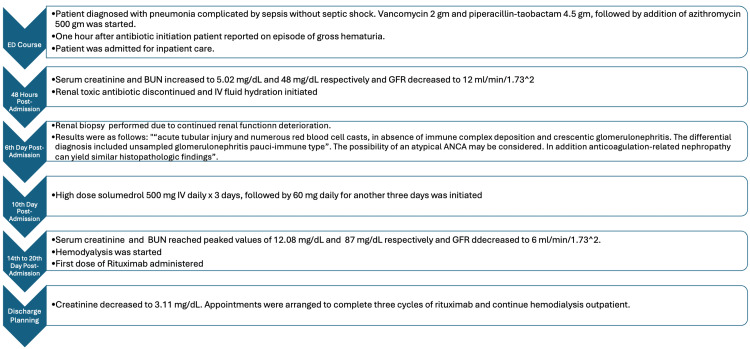
Overview of the patient's clinical course

After initiation of hemodialysis, steroids, and the first dose of rituximab, the patient’s renal function began to improve, and he was discharged with a creatinine level of 3.11 mg/dL, as seen in Table 2. The patient was instructed to continue care in the outpatient setting to complete three cycles of rituximab and hemodialysis. Figure 2 provides an overview of the patient's clinical course. 

## Discussion

This case exemplifies the multifactorial nature of AKI in patients with complex comorbidities, with sepsis being a primary contributor but without neglecting renal toxic antibiotics and pauci-immune-mediated glomerulonephritis as important contributors to the pathophysiology. 

An important consideration for the cause of AKI is the use of renal toxic antibiotics. However, while the combination of vancomycin and piperacillin-tazobactam carries a high risk of AKI, the typical onset of AKI typically occurs eight days after the initiation of therapy [3]. Therefore, the rapid onset of hematuria within one hour after treatment initiation and the increase of creatinine and BUN just 48 hours after treatment initiation make renal toxic antibiotics a less likely culprit of the patient’s presentation.

In this case, while sepsis is considered a potential cause of the patient's presentation, the most likely underlying issue appears to be ANCA-negative pauci-immune glomerulonephritis. Sepsis-related AKI involves complex pathophysiology that is not entirely understood, though it is believed that the inflammatory response plays a crucial role. Histopathological findings in sepsis-mediated AKI often show patchy and heterogeneous tubular cell injury with apical vacuolization but without tubular necrosis, as was observed in our patient [4]. A significant factor in organ dysfunction in sepsis is the widespread activation of neutrophils, which is frequently associated with elevated MPO levels, leading to oxidative stress and tissue damage [5].

However, the rapid and severe deterioration of the patient's renal function, along with the clinical presentation, suggested the possibility of an ANCA-negative pauci-immune glomerulonephritis affecting the unsampled renal parenchyma. Due to the fulminant and rapidly progressive nature of the renal impairment, aggressive immunosuppressive treatment with rituximab was initiated. Following this treatment, the patient's renal function showed significant improvement, supporting the hypothesis that an underlying immune-mediated and systemic inflammatory response was the most likely cause of the patient's condition.

## Conclusions

In conclusion, this case underscores the multifactorial nature of AKI in patients with complex comorbidities, highlighting the challenges in identifying the primary contributors to renal dysfunction. While sepsis emerged as a primary factor due to its association with systemic inflammation and rapid renal deterioration, the potential role of renal toxic antibiotics and ANCA-negative pauci-immune glomerulonephritis could not be overlooked. This case highlights the importance of considering multiple potential etiologies in the differential diagnosis of AKI, especially in the presence of complex clinical presentations. It also emphasizes the need for a comprehensive approach that integrates clinical, laboratory, and histopathological data to guide effective treatment strategies and improve patient outcomes.
